# Analyses of Child and Youth Self-Poisoning Hospitalizations by Substance and Socioeconomic Status

**DOI:** 10.3390/ijerph18137003

**Published:** 2021-06-30

**Authors:** Samantha Pawer, Fahra Rajabali, Alex Zheng, Jennifer Smith, Roy Purssell, Ian Pike

**Affiliations:** 1 BC Injury Research and Prevention Unit, BC Children’s Hospital, Vancouver, BC V6H 3V4, Canada; samantha.pawer@bcchr.ca (S.P.); alex.zheng@bcchr.ca (A.Z.); jsmith@bcchr.ca (J.S.); ipike@bcchr.ca (I.P.); 2 Department of Emergency Medicine, University of British Columbia, Vancouver, BC V5Z 1M9, Canada; roy.purssell@bccdc.ca; 3 British Columbia Drug and Poison Information Centre, BC Centre for Disease Control, Provincial Health Services Authority, Vancouver, BC V5Z 4R4, Canada; 4 Department of Pediatrics, University of British Columbia, Vancouver, BC V6H 3V4, Canada

**Keywords:** self-harm, poisoning, adolescent, socioeconomic status, analgesics, antidepressants

## Abstract

Child and youth self-poisoning is a growing public health issue in many regions of the world, including British Columbia (BC), Canada, where 15–19-year-olds have the highest rates of self-poisoning hospitalizations compared with those of all other ages. The purpose of this study was to identify what substances children and youth commonly used to poison themselves in BC and how socioeconomic status may impact self-poisoning risk. Self-poisoning hospitalization rates among 10–14 and 15–19-year-olds from 1 April 2012 to 31 March 2020 were calculated by substance using ICD-10-CA codes X60-X69 and T36-T65, as well as by socioeconomic status using the Institut National de Santé Publique du Québec’s Deprivation Index. Nonopioid analgesics, antipyretics, and antirheumatics were the most common substances involved, with rates of 27.6 and 74.3 per 100,000 population among 10–14 and 15–19-year-olds, respectively, followed by antiepileptic, sedative–hypnotic, antiparkinsonism, and psychotropic drugs, with rates of 20.2 and 68.1 per 100,000 population among 10–14 and 15–19-year-olds, respectively. In terms of socioeconomic status, rates were highest among 10–19-year-olds living in neighbourhoods with the fewest social connections (243.7 per 100,000 population). These findings can inform poisoning prevention strategies and relevant policies, thereby reducing the number of self-poisoning events among children and youth.

## 1. Introduction

Globally, self-inflicted injuries are the second leading cause of death among 10–24-year-olds [1]. From 2006–2013 in the United States, emergency department visits resulting from attempted suicide or self-harm were highest among 15–19-year-olds, with a rate of 350.7 per 100,000 population [2]. Self-harm, which is a concerning behavior on its own, is a crucial matter to address, as 10–18-year-olds who have previously conducted self-harm are 30 times more likely to die by suicide in the following year compared with what is expected in the general age-matched population [3]. As well, among those who self-harm at 16 years old, there is an increased risk of future mental health concerns, subsequent self-harm, and impending problematic substance misuse [4].

In particular, self-poisoning among children and youth is a significant concern around the world. Among 12–17-year-olds in England, self-poisoning was the most common method of self-harm leading to hospitalization [5]. In the Australian states of New South Wales and Victoria, intentional poisonings among 5–19-year-olds increased by 8.39% annually from 2006–2016 [6], while across the United States, self-poisoning calls to poison centers increased by nearly 300% among 10–15-year-olds from 2000–2018 [7].

Specifically in British Columbia (BC), Canada, 10–14 and 15–19-year-olds were the only age groups that experienced increasing self-poisoning hospitalization rates from 1 April 2009 to 31 March 2017 [8]. Additionally, 15–19-year-olds in BC had the highest rates of self-poisoning hospitalizations compared with all other age groups, with particularly high rates in rural regions of the province that had fewer local mental health services [8]. Nevertheless, many questions remain regarding other sociodemographic characteristics that may affect the risk of self-poisoning among children and youth, as well as what types of substances are commonly involved in these events.

It has been well-established that low socioeconomic status is associated with poorer health outcomes among children and youth, such as by resulting in more severe asthma symptoms and higher rates of obesity [9]. Pertinent to this study, low socioeconomic status has been associated with increased child suicide attempts [10]. For example, it was identified that predictors of self-reported suicide attempts among 15–16-year-olds in 17 European countries included not living with both parents and low socioeconomic status [11]. Similarly, higher incidence of self-harm among 13–18-year-olds in Sweden was linked to parents with lower education or a history of a mental health disorder and single-parent households [12].

Among 15–24-year-old Canadian youth seeking treatment for substance use and mental health issues, a disproportionately high number of individuals expressed concerns about the social determinants of health [13], suggesting that socioeconomic factors influence rates of substance use and mental health issues among this population. However, a knowledge gap exists regarding the extension of this relationship to child and youth self-poisoning in Canada.

To study public health issues in relation to socioeconomic status, the Institut national de santé publique du Québec (INSPQ), a public health institute in Quebec, Canada, developed a neighbourhood deprivation index with six socioeconomic indicators—three material and three social—extracted from Canadian census data [14]. This index was validated in Quebec [15] and used in Canadian research [16], including pediatric studies [17]. Material deprivation indicates a community’s possession of everyday goods and commodities, and social deprivation reflects the social networks of those living within an area [14]. The factors are measured among those aged 15 years and older. The factors related to neighbourhood material deprivation are (1) the proportion of individuals who have not obtained a secondary school diploma, (2) the ratio of employment to population, and (3) average income. The factors related to neighbourhood social deprivation are (1) the proportion of individuals who live alone; (2) the proportion of those who are separated, divorced, or widowed; and (3) the proportion of single-parent families [14].

Understanding how socioeconomic status may relate to self-poisoning rates among children and youth will inform self-poisoning prevention strategies and reduce the number of these events that occur among young people. It is crucial to learn more about what substances children and youth use to poison themselves for the future implementation of effective policies to regulate these specific substances. Accordingly, this study aimed to both determine common substances involved in child and youth self-poisoning hospitalizations in BC and employ the INSPQ deprivation index to investigate how material and social deprivation relate to self-poisoning hospitalizations among this population.

## 2. Materials and Methods

### 2.1. Study Overview

Self-poisoning hospitalization data for 10–19-year-olds in BC from 1 April 2012 to 31 March 2020 was retrospectively described by poisoning substance and with respect to economic and social factors. Hospitalizations were defined as poisoning events leading to a hospital admission for inpatient care. Approval for this study was granted by the University of British Columbia/Children’s and Women’s Health Centre of British Columbia Research Ethics Board (#H13-01321).

Self-harm includes both suicide attempts and non-suicidal self-harm [18]. Although motivations for self-harm can be difficult to confirm, it has been reported that up to 36% of individuals who self-harm have suicidal intentions [19]. In the present study, suicide attempts could not be differentiated from non-suicidal self-harm because cases were coded broadly as self-harm events. Therefore, both suicide attempts and non-suicidal self-harm are captured in our self-poisoning rates.

### 2.2. Data Sources and Characteristics

From 1 April 2012 to 31 March 2020, inclusive, acute hospital separation data for those admitted to hospital was obtained from the Discharge Abstract Database (DAD), BC Ministry of Health. This dataset included demographic, administrative, and clinical factors by case, including sex, age, primary cause of hospitalization, poisoning substance, and residential dissemination area (DA; a region where roughly 400–700 live). Cases without a recorded sex were excluded from analyses by sex. The *International Statistical Classification of Diseases and Related Health Problems Canadian version 10* (ICD-10-CA) was used to define poisoning hospitalizations [20], including poisoning by adverse effects of and underdosing of substances and toxic effects of substances. The present study included cases with codes X60–X69 that encompass intentional self-poisonings, as well as codes T36–T65 to identify the poisoning substance involved, such as poisoning by, adverse effect of, and underdosing of nonopioid analgesics, antipyretics, and antirheumatics (T39) and toxic effect of alcohol (T51). Population data by DA were obtained from the Statistics Canada 2011 and 2016 Census Profiles [21,22].

The INSPQ 2011 and 2016 neighbourhood deprivation indices were used as a proxy for socioeconomic status to explore how the material and social wealth of a neighbourhood were related to rates of self-poisoning hospitalizations among children and youth. The 2016 deprivation indices and Census population were used primarily; however, in the absence of a DA in 2016 because of DA reconfigurations, the 2011 deprivation indices and 2011 Census population were applied. When no 2011 or 2016 information was available, cases were excluded. Material and social deprivation indices are divided into five quintiles, with each quintile representing approximately 20% of the BC population. Each DA is assigned a material quintile and a social quintile. Quintile 1 represents the least deprived DAs, while Quintile 5 represents the most deprived DAs.

### 2.3. Data Analysis

Self-poisoning hospitalization rates per 100,000 population were calculated by material and social deprivation indices. First, the average number of annual cases within each quintile was determined, using cases among residents in DAs assigned to each quintile. Second, the 10–19-year-old population for each quintile was calculated by summing the number of residents in every DA assigned to each quintile. Third, the average number of annual cases for each quintile was divided by the 10–19-year-old population for that quintile. Finally, this value was multiplied by 100,000 to obtain a self-poisoning hospitalization rate per 100,000 population for each of the five material and social quintiles.

Similarly, self-poisoning rates by substance were calculated using the total number of cases related to each substance, divided by the provincial population of 10–14 or 15–19-year-olds throughout the study period, and multiplied by 100,000. For each rate, Wald 95% confidence intervals were calculated, and rates were determined to be significantly different when the 95% confidence intervals did not overlap.

## 3. Results

### 3.1. Overview of Self-Poisoning Hospitalizations

From 1 April 2012 to 31 March 2020, there were 4962 self-poisoning hospitalizations among 10–19-year-olds in BC (120.6 per 100,000 population). This included 97 males (10.0 per 100,000 population) and 1039 females (113.1 per 100,000 population) aged 10–14 years, as well as 776 males (67.3 per 100,000 population) and 3047 females (283.6 per 100,000 population) aged 15–19 years. Within each age group, rates were significantly higher among females relative to males. There were fewer than 5 patients (<0.06%) excluded from analyses due to missing sex data.

### 3.2. Self-Poisoning by Substance

Nonopioid analgesics, antipyretics, and antirheumatics (e.g., acetaminophen and ibuprofen) were the most common substances used by 10–14 and 15–19-year-olds admitted to BC hospitals for self-poisoning, with rates of 27.6 and 74.3 per 100,000 population, respectively. The second most common substances were antiepileptic, sedative–hypnotic, antiparkinsonism, and psychotropic drugs (e.g., antidepressants), with rates of 20.2 per 100,000 population among 10–14-year-olds and 68.1 per 100,000 population among 15–19-year-olds (Table 1). Other substances resulting in self-poisoning hospitalizations among 10–19-year-olds included unspecified drugs, medicaments, and biological substances (e.g., appetite depressants); other drugs acting on the autonomic nervous system (e.g., certain vasoconstrictors); narcotics and psychodysleptics (e.g., heroin); other and unspecified chemical and noxious substances (e.g., detergents and paint); organic solvents and hallogenated hydrocarbons and their vapours (e.g., petroleum); alcohol; and gases and vapours (e.g., carbon monoxide; Table 1). All substances not included in these categories were grouped as other substances.

### 3.3. Self-Poisoning by Material and Social Deprivation

There were 180 self-poisoning hospitalizations excluded (3.6% of hospitalizations) due to missing DA or quintile information.

**Figure 1:**
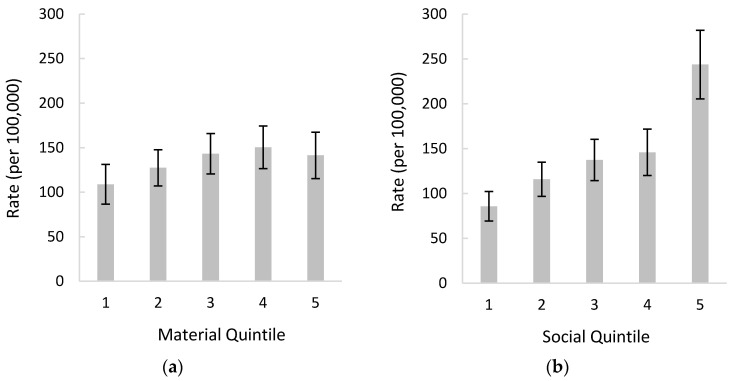
Self-poisoning hospitalization rates per 100,000 population in British Columbia (BC) among 10–19-year-olds, 1 April 2012 to 31 March 2020, by: (**a**) material quintile; (**b**) social quintile. Error bars display 95% confidence intervals.

Generally, there was no relationship between self-poisoning hospitalization rates and material deprivation among 10–19-year-olds. The largest difference was between the first (least deprived) and fourth (second most deprived) material quintiles, as the rate increased from 108.9 to 150.4 per 100,000 population but was not significantly different (Figure 1a). A more evident increase in self-poisoning rates was observed with increasing social deprivation, as there was a 2.8-fold increase in self-poisoning hospitalization rates from the first (least deprived) to fifth (most deprived) social quintiles (85.8 to 243.7 per 100,000 population). Notably, the greatest self-poisoning hospitalization rates were among those in the fifth (most deprived) social quintile (Figure 1b). There was a 1.7-fold increase in self-poisoning hospitalization rates from the fourth to fifth social quintiles (145.9 to 243.7 per 100,000 population), which was the only significant increase between successive quintiles for either material or social deprivation.

**Figure 2:**
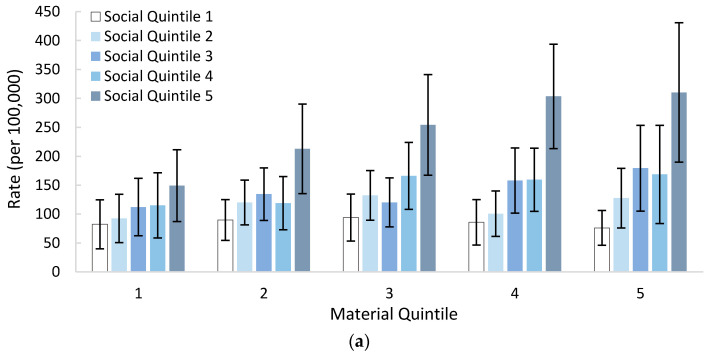

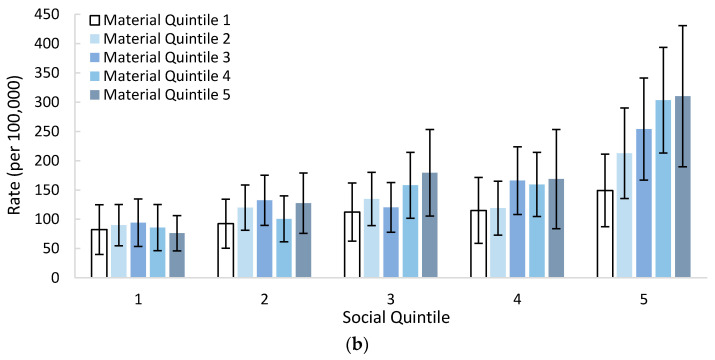
Self-poisoning hospitalization rates per 100,000 population in BC among 10–19-year-olds, 1 April 2012 to 31 March 2020, by: (**a**) social quintile controlling for material quintile; (**b**) material quintile, controlling for social quintile. Error bars display 95% confidence intervals.

Controlling for material deprivation, self-poisoning hospitalization rates among 10–19-year-olds generally increased with social deprivation. Within each material quintile, there were large increases from the fourth to fifth social quintiles (1.2- to 2.2-fold increases in magnitude; Figure 2a). After controlling for social deprivation, self-poisoning hospitalization rates more reliably increased with material deprivation as social deprivation increased (Figure 2b). When considering both material and social deprivation, 10–19-year-olds with the highest self-poisoning hospitalization rate were those residing in neighbourhoods with the lowest material and social wealth (quintile 5), having a rate of 310.2 per 100,000 population.

## 4. Discussion

Self-poisoning among children and youth has been identified as a critical public health concern in many areas across the globe, for example, in countries such as England [5] and Iran [23], as well as throughout North America [7,24]. Previous work has demonstrated that BC is no exception [8,25]. In terms of demographics, studies from both the United States and Newfoundland and Labrador, Canada, identified that older teenagers and females have higher incidences of self-poisoning compared with younger teenagers and males [7,24]. We identified similar trends, with 15–19-year-old females having the highest rates of self-poisoning hospitalizations for every poisoning substance included in this study. While other jurisdictions had previously determined common substances used by youths who intentionally poisoned themselves, there was little similar literature using a Canadian population, which is essential for designing and implementing successful poisoning prevention strategies geared towards subpopulations that would benefit most.

We found that among both 10–14 and 15–19-year-olds, the highest rates of self-poisonings involved nonopioid analgesics, antipyretics, and antirheumatics. This is parallel to findings from other studies. In England, poisonings among 10–24-year-olds most commonly involved paracetamol (also known as acetaminophen—a nonopioid analgesic) [26], while in the United States, 10–25-year-olds who self-poisoned most commonly used over-the-counter analgesics [27]. Additionally, in the Czech Republic, 9–18-year-olds who self-poisoned with a single substance most commonly used anti-inflammatory/antirheumatic products [28]. Our findings were also similar to that of previous studies in that we identified that antiepileptic, sedative–hypnotic, antiparkinsonism, and psychotropic drugs were the second most common class of substances that resulted in self-poisoning hospitalizations, while antidepressants (a category of psychotropic drugs) and sedative/hypnotics were found to be the second and third most common substances used in the United States [27].

It is interesting to note that after nonopioid analgesics, antipyretics, and antirheumatics, and antiepileptic, sedative–hypnotic, antiparkinsonism, and psychotropic drugs, we observed a significant drop in self-poisoning hospitalization rates among 10-19-year-olds resulting from all other substances. A potential explanation for this could be having easy access to nonopioid analgesics, antipyretics, and antirheumatics, and antiepileptic, sedative–hypnotic, antiparkinsonism, and psychotropic drugs. Many nonopioid analgesics, antipyretics, and antirheumatics can be purchased over the counter, while ADHD psychostimulants and antidepressants (both categorized as antiepileptic, sedative–hypnotic, antiparkinsonism, and psychotropic drugs) were among the most commonly prescribed medications for 6–24-year-olds in Canada from 2007 to 2011 [29].

High rates of self-poisonings involving nonopioid analgesics, antipyretics, and antirheumatics are particularly concerning, as over-the-counter analgesics were linked to 37.3% of poisoning-related attempted suicides resulting in serious outcomes among 10–25-year-olds in the United States [27], and prior poisoning-related hospitalization due to nonopioid analgesics in Denmark significantly increased one’s risk of suicide later in life [30].

Given that there are such high rates of self-poisoning hospitalizations resulting from nonopioid analgesics, such as acetaminophen and ibuprofen, it is critical that this issue be addressed. A potential method of prevention is for physicians to discuss the risks of over-the-counter medications with children and youth or encourage their parents to do so. Drug use is reduced by half among young people who are educated about these risks by their parents [31]. Still focusing on the most common substances linked with self-poisonings, the United Kingdom observed reduced self-poisoning rates after limiting the maximum allowable quantity of acetaminophen per package [32]. Self-harm is often an impulsive act [33], which could explain why smaller quantities of immediately available drugs result in lower rates of self-poisoning. This makes limiting the allowable quantity of over-the-counter pain medications another potential solution for other jurisdictions to adopt in order to aid in the effort to reduce rates of children and youth self-poisoning.

Specifically in BC, current self-harm prevention resources available to children and youth include online platforms advising individuals to share their thoughts with trusted adults, as well as the Kids Help Phone and YouthinBC Online Chat. While helpful, these services only benefit those who seek help, and social support predicts mental health help-seeking behaviour among adolescents [34]. By linking lower social support with increased risk of untreated mental health concerns, this association provides insight into our finding regarding high self-poisoning rates among 10–19-year-olds residing in neighbourhoods with limited social connections. Perhaps these individuals have an elevated risk of self-poisoning because they are unlikely to seek help for mental health concerns. This is supported by findings that child and youth self-poisoning hospitalizations were highest in BC regions with poor access to mental health services [8]. Alternatively, some young people may not have the social support required to utilize these services. Given that our findings demonstrated that low social connectedness is a driving factor for high rates of self-poisoning hospitalizations, yet material wealth made little difference, this highlights the importance of social support and connectedness for successfully reducing self-harm rates among children and youth. Recently, efforts were made in the province to help BC children and youth to obtain mental health, substance use, and other services in a manner that fosters professional and peer support.

Launched in May 2021, the Foundry BC app is available for 12–24-year-olds residing in BC, offering confidential online mental health, substance use, and other services at no charge [35]. Services range from drop-in virtual counselling appointments to peer support and youth groups. Although this service is helpful for improving access to mental health care, it is similar to existing resources in that it may not necessarily benefit youth who are not seeking help. Additionally, the Foundry BC app encourages caregiver involvement to increase recovery rates, which may be a barrier for children and youth with few social connections. Although this app is crucial for increasing accessibility to important services for young people across BC, due to its novelty, its efficacy in reducing self-harm rates has not yet been explored.

An alternative child and youth self-harm prevention strategy is to expand the role of public health. The Signs of Self-Injury (SOSI) Program is a budget-friendly workshop that can be implemented in secondary schools, aiming to reduce self-harm events [36]. This program aligns with the BC Coroner’s recommendation to incorporate emotional learning and well-being strategies into the school setting [37] while reaching a wide audience of children and youth across the province, regardless of their socioeconomic status. The first part of SOSI is for school staff, providing them with advice for reacting to self-harm disclosures and non-suicidal self-injury psychoeducational resources, preparing them to identify signs among students. The second aspect of the program includes a video and several video scenarios to educate students about non-suicidal self-injury, followed by a question period and discussion about local resources. SOSI increases knowledge among students and faculty about non-suicidal self-injury and improves the preparedness of students to seek help [36]—a key issue for those residing in neighbourhoods with low social support [34]. Although SOSI is not specifically focused on self-poisoning prevention, the implementation of this program in schools may reduce all types of self-harm injuries among children and youth, including self-poisoning. Self-harm is less likely among BC youth who feel their thoughts are heard [38], which further supports strategies like the SOSI Program that encourage open dialogue.

Although this study is novel in using a Canadian sample of children and youth to identify common substances used for self-poisoning, as well as socioeconomic risk factors for self-poisoning hospitalizations, it does have limitations. Social and material quintiles were assigned to each DA, since data were not available at an individual level. Therefore, an ecological fallacy is possible where an association found for an aggregate area is applied to individuals. However, evidence has shown that neighbourhood effects often reflect individuals’ conditions [39]. Using administrative data from the DAD has limitations, as it determines which codes are specified, meaning we were unable to obtain further information pertaining to the circumstances or specifics of each substance. Since the DAD is an administrative dataset, the availability of variables collected is also very limited. It may have been beneficial to conduct more complex analyses regarding individual or family risk factors that might affect self-poisoning behavior; however, no such information could be extracted from the DAD. Another limitation is that the INSPQ deprivation index uses Statistics Canada data, which, based on the definition of a census family, recognize single-parent families as those with children who are biological, step, or adopted and live in the same dwelling as the parent, or grandparent if no parents are present [40], thus excluding support from outside the household.

While our findings have made progress towards identifying the sociodemographic groups at highest risk for self-poisoning, as well as what substances BC children and youth most often choose when intentionally poisoning themselves, future research could focus on how ethnicity and culture might impact child and youth self-poisoning rates. Another approach may be to further explore the effect that gender (as opposed to sex) has on self-poisoning rates. We conducted analyses only by sex, although it has previously been found that 13–17-year-olds who are transgender or identify their gender as “other” had higher rates of self-harm ideation compared with cis-gendered agemates [41]. Similar to the idea that children and youth in neighbourhoods with low social connectedness may have high self-poisoning rates due to challenges with seeking help, another study found that non-binary 16–25-year-olds assigned male sex at birth had poor help-seeking behavior for depression and anxiety [42]. Given these connections, future studies looking at self-poisoning rates by gender may help refine the characteristics of youth populations at high risk of self-harm. In addition, our analyses had an end date of 31 March 2020, which was just 20 days after the World Health Organization characterized COVID-19 as a pandemic [43]. It would be useful for future studies to assess the impact that the COVID-19 pandemic has had on rates of child and youth self-harm, or specifically self-poisoning. Such follow-up work would provide further insight regarding risk factors for self-poisoning to ultimately inform prevention practices and policies moving forward.

## 5. Conclusions

In BC, 10–19-year-old children and youth have high rates of self-poisoning hospitalizations, particularly among 15–19-year-old females. From 1 April 2012 to 31 March 2020, the most common classes of substances that resulted in these hospitalizations included nonopioid analgesics, antipyretics, and antirheumatics, as well as antiepileptic, sedative–hypnotic, antiparkinsonism, and psychotropic drugs. Additionally, it was determined that in terms of socioeconomic status, 10–19-year-olds living in the least socially connected neighbourhoods had significantly higher rates of self-poisoning hospitalizations relative to those living elsewhere. These findings can help inform self-poisoning prevention strategies, for example, by encouraging policies to be put in place to limit access to large quantities of over-the-counter pain medications and promoting regulation of prescription antidepressant and anxiety medications. Additionally, to help reach children and youth living in all regions of the province, regardless of socioeconomic status, implementing public health solutions, such as the SOSI Program, may be beneficial. In combination with these strategies, reducing social inequities might also decrease self-poisoning rates among children and youth.

## Figures and Tables

**Table 1:** Rates per 100,000 population, with 95% confidence intervals, for self-poisoning hospitalizations, 1 April 2012 to 31 March 2020, by poisoning substance, sex, and age group.

Age Group	Substance	Male	Female	Overall
10–14	Nonopioid analgesics, antipyretics, and antirheumatics	2.9 (1.8–4.0)	53.4 (48.7–58.2)	27.6 (25.2–30.0)
	Antiepileptic, sedative–hypnotic, antiparkinsonism, and psychotropic drugs	3.5 (2.3–4.7)	37.7 (33.7–41.6)	20.2 (18.1–22.2)
	Unspecified drugs, medicaments, and biological substances	1.4 (0.7–2.2)	9.4 (7.4–11.3)	5.3 (4.3–6.3)
	Other drugs acting on the autonomic nervous system	0.7 (0.2–1.3)	3.6 (2.4–4.8)	2.1 (1.5–2.8)
	Narcotics and psychodysleptics	0.2 (0.0–0.5)	1.7 (0.9–2.6)	1.0 (0.5–1.4)
	Other and unspecified chemical and noxious substances	0.5 (0.1–1.0)	3.4 (2.2–4.6)	1.9 (1.3–2.5)
	Organic solvents and hallogenated hydrocarbons and their vapours	0.7 (0.2–1.3)	1.4 (0.6–2.2)	1.1 (0.6–1.5)
	Alcohol	0.0 (0.0–0.0)	1.0 (0.3–1.6)	0.5 (0.2–0.8)
	Gases and vapours	0.0 (0.0–0.0)	0.5 (0.1–1.0)	0.3 (0.0–0.5)
	Other substances ^	0.0 (0.0–0.0)	1.1 (0.4–1.8)	0.5 (0.2–0.9)
15–19	Nonopioid analgesics, antipyretics, and antirheumatics	24.9 (22.0–27.8)	127.2 (120.5–134.0)	74.3 (70.7–77.9)
	Antiepileptic, sedative–hypnotic, antiparkinsonism, and psychotropic drugs	28.9 (25.8–32.0)	110.2 (103.9–116.5)	68.1 (64.7–71.5)
	Unspecified drugs, medicaments, and biological substances	4.9 (3.6–6.1)	16.0 (13.6–18.4)	10.2 (8.9–11.6)
	Other drugs acting on the autonomic nervous system	2.0 (1.2–2.8)	10.4 (8.5–12.4)	6.1 (5.0–7.1)
	Narcotics and psychodysleptics	2.9 (1.9–3.8)	5.4 (4.0–6.8)	4.1 (3.2–4.9)
	Other and unspecified chemical and noxious substances	1.0 (0.5–1.6)	5.6 (4.2–7.0)	3.2 (2.5–4.0)
	Organic solvents and hallogenated hydrocarbons and their vapours	1.0 (0.5–1.6)	3.0 (1.9–4.0)	2.0 (1.4–2.6)
	Alcohol	1.2 (0.6–1.9)	2.9 (1.9–3.9)	2.0 (1.4–2.6)
	Gases and vapours	0.3 (0.0–0.7)	0.8 (0.3–1.4)	0.6 (0.3–0.9)
	Other substances ^	0.2 (0.0–0.4)	2.0 (1.2–2.9)	1.1 (0.6–1.5)

^ Other substances include systemic antibiotics; systemic anti-infectives and antiparasitics; hormones and their synthetic substitutes and antagonists, not elsewhere classified; anesthetics and therapeutic gases.

## Data Availability

Restrictions apply to the availability of these data. Hospitalization data were obtained from Discharge Abstract Database, BC Ministry of Health; rate data are available from F.R. upon request.
